# Distinct fragmentation patterns of circulating viral cell-free DNA in 83,552 non-invasive prenatal testing samples

**DOI:** 10.20517/evcna.2021.13

**Published:** 2021-09-30

**Authors:** Jasper Linthorst, Matthijs R. A. Welkers, Erik A. Sistermans

**Affiliations:** ^1^Department of Human Genetics and Amsterdam Reproduction & Development research institute, Amsterdam UMC, Vrije Universiteit Amsterdam, Amsterdam 1081 BT, The Netherlands.; ^2^Department of Medical Microbiology & Infection Prevention, Amsterdam UMC, Amsterdam 1081 BT, The Netherlands.

**Keywords:** Virome, fragmentomics, DNA degradation, epigenetics, viral cell-free DNA, NIPT

## Abstract

**Aim:**

The fragmentation characteristics of cell-free DNA (cfDNA) are informative biomarkers in liquid biopsies, including non-invasive prenatal testing (NIPT), as they provide insights into the origins of the cfDNA. Viral infections by DNA viruses can contribute to the available cfDNA in these samples. Here, we characterize the fragment size distribution of viral cfDNA fragments obtained from available anonymous NIPT samples.

**Methods:**

A viral database of 224 DNA viruses was generated from the NCBI RefSeq viral database. Paired-end cfDNA sequencing reads from 83.522 NIPT samples that did not map to any of the human chromosomes, or mitochondrial DNA of the human reference genome build GRCh38 (excluding alternative and unplaced contigs) were remapped to the generated viral database. Reads mapping to the 14 most abundant DNA viruses were selected, and fragment size distributions were analyzed in detail.

**Results:**

Distinct fragmentation patterns were identified for several DNA viruses, most likely due to differences in viral tropism, chromatinization (binding of nucleosomes), and the topology of the viral DNA. In high viral load parvo B19 positive samples, the fragment size distribution differed between samples, potentially reflecting the state of the infection.

**Conclusion:**

These findings outline the potential for liquid biopsies to elucidate the dynamics behind the viral infection, which may potentially have various clinical applications. Our data provide preliminary insights on the use of fragmentomics of viral cfDNA to distinguish between reactivation, reinfection, and primary infection and monitoring the state of viral infections.

## INTRODUCTION

Non-invasive prenatal testing (NIPT) in pregnant women can be performed by untargeted whole genome sequencing of cell-free DNA (cfDNA). While cfDNA predominantly stems from chromosomal DNA of apoptotic blood cells^[1]^, small amounts of viral cfDNA can also be detected in NIPT samples^[2]^. As cfDNA is not sheared before sequencing, the insert size of a paired-end sequencing read can be used to determine the actual size of the cfDNA fragments^[3]^. In maternal blood plasma, most cfDNA fragments of fetal (placental) origin are 147bp (the size of a nucleosome), while most fragments of maternal origin are 166bp (the size of a nucleosome + the h1 linker histone, named a chromatosome)^[4]^. During genome deconstruction of apoptotic cells, nucleosomes impede endonuclease activity causing most chromosomal cfDNA fragments to be the size of approximately a nucleosome. This characteristic size distribution is predominantly driven by the nucleosome occupancy of the underlying apoptotic cells. This is confirmed by the different size distribution of cell-free mitochondrial DNA, which is known to be free of nucleosomes and does not show any increase in nucleosome-sized fragments^[1]^. These molecular characteristics of cfDNA, termed fragmentomics, are also used in liquid biopsies (such as NIPT) to estimate the fraction of placenta-derived DNA in a sample^[5]^ or in cancer diagnosis to detect marks of aberrant epigenetic regulation^[6]^.

Infectious viral DNA is packaged in virions in which the genome is protected by a capsid structure free of nucleosomes^[7]^. Upon infecting a cell, the viral DNA is rapidly loaded with histones to suppress viral gene expression^[7]^. Although this form of “epigenetic silencing” inhibits viral gene expression and initially prevents the spread of the infection, it also enables the virus to evade the host immune system and establish a latent infection. Studies have also shown that the nucleosome positioning on viral DNA within the nucleus of infected cells is dynamic, non-random, and presumably plays a vital role in the lifecycle of the virus^[7-9]^.

Viral cfDNA fragments, as sequenced by cfDNA sequencing, are naturally degraded short fragments of DNA. They are expected to result from either (latently) infected apoptotic cells or degraded virions. Therefore, the detection of viral sequences in cfDNA is no direct evidence of a productive viral infection. However, the size distribution of the viral cfDNA fragments may provide clues as to whether (or what fraction of) the originating viral genomes were infectious or not. This is based on the assumption that nucleosome-bound viral cfDNA stems from apoptotic infected cells, while nucleosome-free viral cfDNA stems from virion DNA.

Previously it was shown that both virion-associated Parvovirus B19 and non-infectious “naked” Parvovirus B19 DNA could be detected from blood samples of asymptomatic blood donors. In addition, exogenous treatment with endonucleases could be used to distinguish infectious from non-infectious parvovirus B19 DNA^[10]^. Similarly, for Epstein-Barr virus (EBV), it was shown that both “naked” and virion-associated DNA could be detected in plasma and that depending on the type of EBV-associated disease, the combination of the two differed^[11]^. It has also been shown that the fragment size distribution of EBV is an informative biomarker for the early detection of nasopharyngeal cell carcinoma (NPC) from liquid biopsies using cfDNA sequencing^[12,13]^. Longer, assumed nucleosome bound, circulating EBV fragments were more frequently observed in NPC cases than non-NPC cases. In a different study^[14]^, circulating cytomegalovirus cfDNA was shown to be exceptionally fragmented, explaining discrepancies in viral load estimates between PCR and NGS-based detection methods, as in PCR, the primers could be too widely spaced to detect the short fragments. Furthermore, the median CMV fragment length was shorter in NIPT samples than cfDNA from transplant patients who had higher viral loads on average. One explanation for these differences was that they were suspected to arise from differences in the epigenetic state of the circulating CMV or the state of reactivation.

Here we explore the fragment size distributions of the 14 most abundant DNA viruses in our dataset. The cfDNA fragments were extracted from 83.552 NIPT samples.

## METHODS

### Data

De-identified cell-free paired-end cfDNA sequencing data was used from NIPT analyses handled by the Amsterdam UMC between June 2018 and December 2020, and all samples were processed using the VeriSeq method (Illumina, San Diego, USA). Upon isolation of cfDNA, samples were paired-end sequenced using 36bp on an Illumina NextSeq500. Raw base call files were de-multiplexed, and adapters were trimmed using bcl2fastq (2.17.1.14), resulting in, on average, ~22.5 million reads per sample.

### Viral database construction and grouping of viral genomes

From the NCBI RefSeq viral database, 224 human host DNA viruses (excluding RNA retroviruses) were selected [Supplementary Table 1] and combined into a single viral reference dataset. We applied the DUST algorithm as implemented in the meme-suite^[15]^ (version 5.3) with default settings to mask regions of low-complexity sequence to prevent spurious alignments. Highly identical reference assemblies were grouped using the NCBI taxonomy database and assigned to specific virus groups [Supplementary Table 1].

In order to query the database, the ETE3 python package^[16]^ was used. Next, distances between reference assemblies were computed using Mash^[17]^, and ambiguous alignments were inspected using the XA tag reported by BWA^[18]^. Finally, groups of highly identical genomes were linked to knots in the taxonomic tree and labeled accordingly. Knots were chosen such that the number of ambiguously positioned reads that exceed group boundaries would be minimized.

### Extraction and alignment of unmapped reads

Reads that did not map to the human genome (build GRCh38; excluding alt, random and unplaced sequence) were extracted from the samples using Samtools (-f 4)^[19]^. All unmapped reads were subsequently aligned to our database of viral genomes using BWA mem^[20]^ (version 0.7.17), and duplicate reads were marked (and later discarded in further analyses) using Samblaster^[21]^.

Viruses for which more than 100 paired-end reads could be mapped in the correct orientation were used to model fragment-size distributions [Supplementary Table 2]. Fragment sizes were derived from the “template length” field as calculated by pysam (https://github.com/pysam-developers/pysam). There was no correlation between the size of the viral genome and the number of aligned reads (Spearman *r *= 0.24, *P *= 0.33; Pearson *r *= 0.24, *P *= 0.34).

### Fetal fraction

The fraction of fetal cfDNA within the sequenced maternal plasma was calculated using the SeqFF method^[22]^.

### Data processing

All non-duplicate reads aligned to our viral reference database were merged into a single cram^[23]^. Then, fragment sizes were calculated, and reads were linked to the input samples through the attached read group tags. Data analysis and plotting were performed using Python and the libraries Pysam, Seaborn (0.11.0), Pandas (1.1.1), Numpy (1.19.5), and Scipy (1.6.0).

## RESULTS

**Figure 1:**
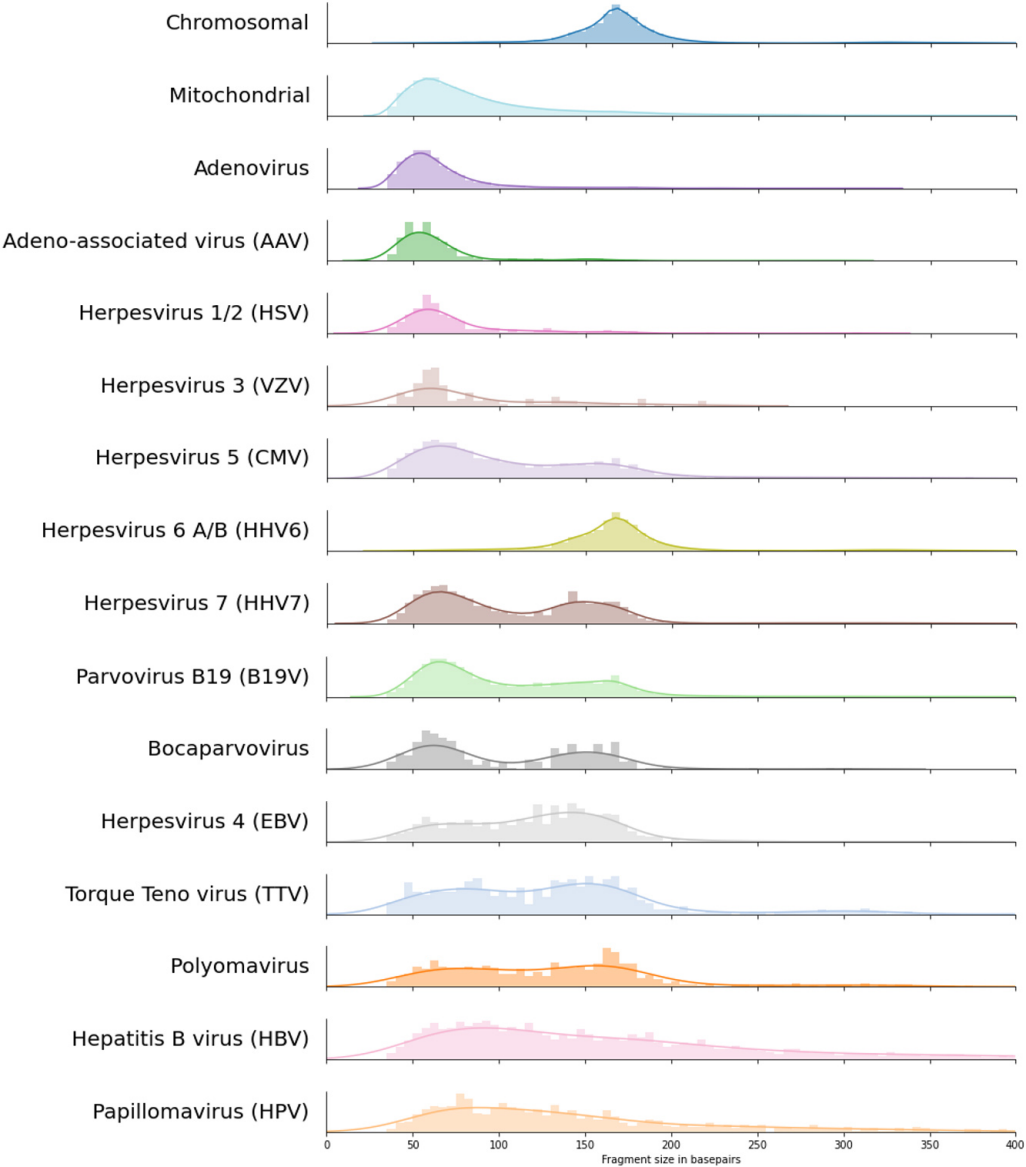
cfDNA fragment length density distributions for the 14 viruses with most paired viral reads with on top the typical human cfDNA fragment length density distribution. Colors are transferred from Figure 2, and viruses are sorted by the total number of paired viral reads that were used to estimate the fragment length densities; bin sizes are adjusted according to the total number of observed paired reads. cfDNA: Cell-free DNA.

**Figure 2:**
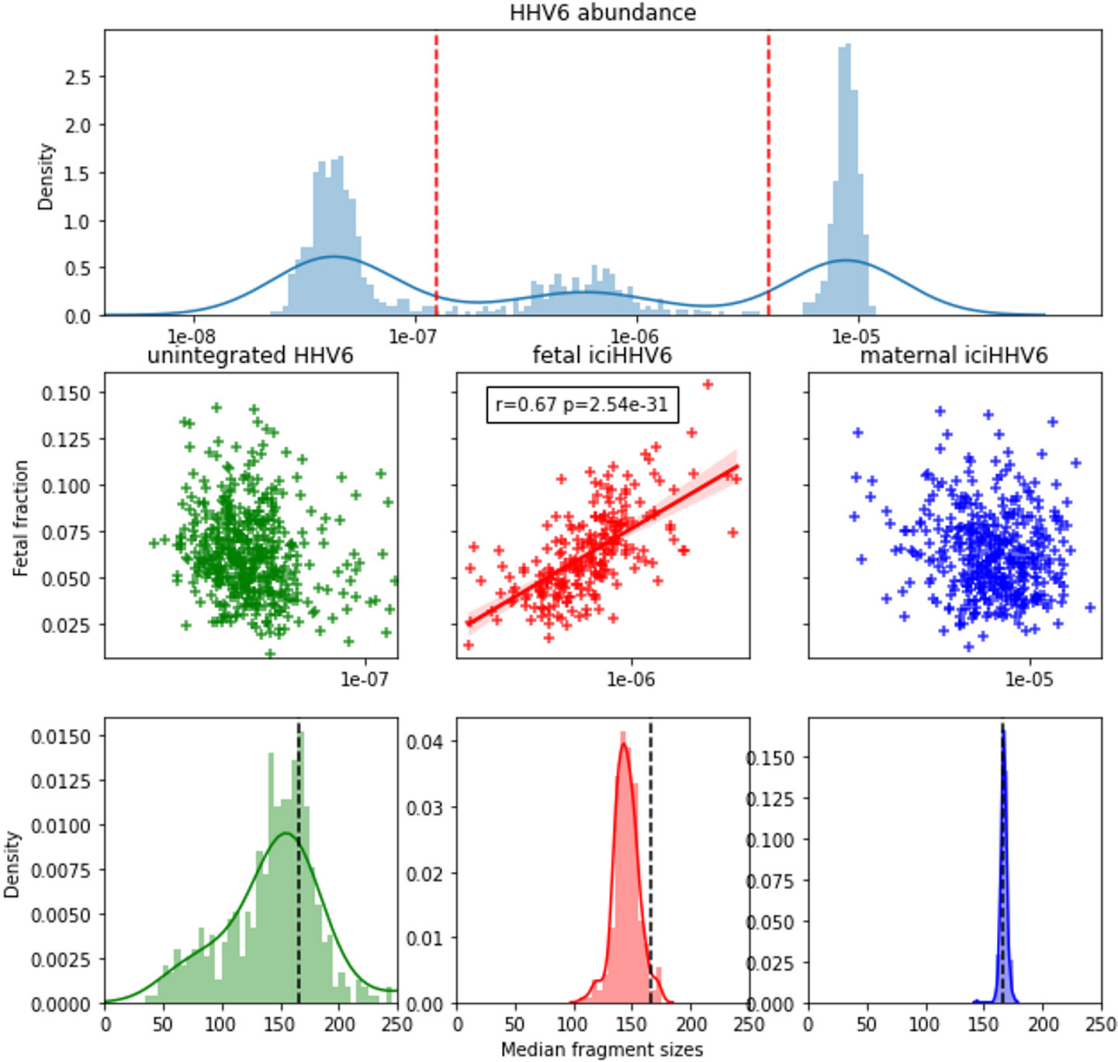
The abundance of HHV6 distinguishes (paternally inherited) fetal and maternal iciHHV6 and shows differing characteristic fragment size distributions. (A) Distributions of HHV6 abundance expressed as the number of viral HHV6 fragments divided over the total number of chromosomal fragments across all HHV6 positive samples on the x-asix. The y-axis represents a histogram (light blue bars) paired with kernel density estimation (dark blue line). Red dashed vertical lines indicate how samples are grouped into low (unintegrated/inherited HHV6; *n *= 479), intermediate (paternally inherited fetal iciHHV6; *n *= 226), and high (maternal iciHHV6; *n *= 441) abundance. (B) Scatter plots indicating the correlation between the calculated fetal fraction of a NIPT sample (y-axis) and the HHV6 abundance (x-axis). A significant correlation is only observed between the calculated fetal fraction and fetal iciHHV6 (red). (C) Different size distributions are observed for the three groups. Typical size distributions are observed with a peak of 143bp for fetal chromosomal cfDNA (red) and maternal cfDNA (blue). The presumed non-iciHHV6 fragments exhibit an increased frequency of short (50bp-100bp) cfDNA fragments. The dashed black line in all three figures indicates the typical 166bp fragment size of chromosomal cfDNA. HHV6: Human herpesvirus 6; iciHHV6: inherited chromosomally integrated HHV6 viruses; NIPT: non-invasive prenatal testing; cfDNA: cell-free DNA.

A total of 139,573 read pairs of suspected viral origin were detected across 13,314 NIPT samples of unique pregnancies. Per NIPT sample, we generally detect a small number of reads of suspected viral origin (for details, see Supplementary Table 2). However, by pooling this data, we obtained sufficient fragments to investigate the fragment size distributions of 14 viral species and compared it to the size distribution of human chromosome and mitochondrial-derived cfDNA [Figure 1]. Except for human herpesvirus 6 (HHV6), the fragment size distributions of all DNA viruses were highly distinct from that of chromosomal cfDNA. For adenovirus, adeno-associated virus (AAV), herpes simplex virus (HSV), and varicella-zoster virus (VZV), only very short viral cfDNA fragments were observed with no increase in the number of nucleosome-sized fragments; comparable to what is seen for mitochondrial cfDNA. The fragment size distribution of HHV6 seemed to exactly overlap with that of chromosomal cfDNA, indicating that the majority of the sequenced HHV6 fragments originated from the inherited chromosomally integrated HHV6 viruses (iciHHV6)^[24]^, which is known to be present on 0.5% of human alleles. The abundance of HHV6 fragments across all NIPT samples followed a trimodal distribution [Figure 2A] which most likely reflects that the iciHHV6 genotype of the mother and fetus contributes most of the HHV6 reads in samples with a high or intermediate HHV6 abundance. In samples with intermediate HHV6 abundance, the abundance correlated with the calculated fetal fraction, in line with germline integration of HHV6 on the paternal allele in the genome of the fetus [Figure 2B]. Although this indicates that genotyping paternally inherited fetal iciHHV6 from NIPT sequencing seems feasible, we did not study maternally inherited iciHHV6 or potential HHV6 integration copy-number differences. In addition, the modes of the fragment size distributions of intermediate and high HHV6 abundant samples exactly overlapped with the size distributions of fetal (peaks around the size of a single nucleosome: 143bp) and maternal (peaks around the size of a chromatosome: 166bp) chromosomal cfDNA respectively [Figure 2C]. The fragment size distribution of samples with a low abundance was significantly different (Kolmogorov Smirnoff test, *P *= 3.74e-17) from the size distribution of samples with an intermediate HHV6 abundance and contained more HHV6 fragments in the size range of 50-100bp. Indicative of the fact that in low abundant HHV6 samples, viral fragments were observed that originated from unchromatinized not chromosomally integrated viral cfDNA, possibly from degraded HHV6 virions.

Further direct evidence for these results follows from the viral chimeric reads, for which one end aligns to the human genome and the other to a viral genome. For many of these reads, we found that the end that maps to the human genome has an extreme GC content (> 85%) which are predominantly aligned to a G-homopolymer on chromosome 2. This resulted in a very different GC content between the human and viral end of the two pairs. The origin of this bias is unclear but may be caused by errors in sequencing of, for example, very short or perhaps single-stranded cfDNA fragments. After excluding the chimeric reads for which the human end had a GC content of above 85%, we found 113 reads for which one end mapped to the human genome and the other end mapped to HHV6. These reads all originated from samples with an intermediate (5) and high (108) abundance of HHV6. However, as HHV6 is also known to integrate its genome into the telomeres of infected somatic cells, we cannot exclude that such reads cannot be observed from low-abundant HHV6 samples. Furthermore, as HHV6 preferably integrates into the telomeres of human chromosomes, it is somewhat complicated to accurately position them on the human genome, as the telomeric sequence is highly repetitive and occurs on all chromosomes. Presumably, for this reason, we found that the 113 chimeric HHV6 reads were distributed across almost all human chromosomes.

**Figure 3:**
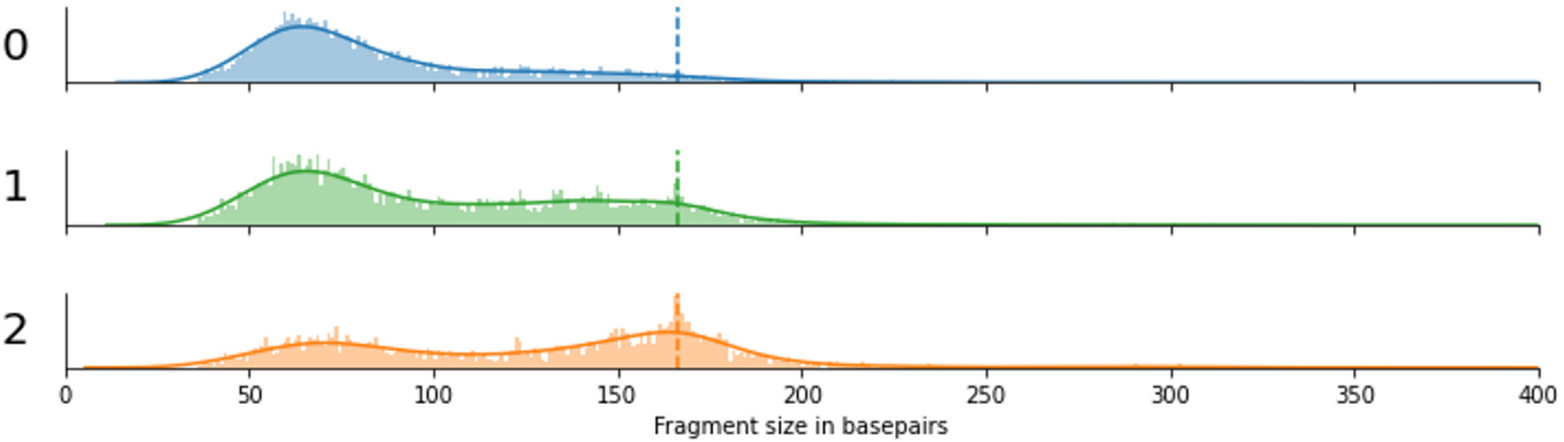
Fragment size distributions of three independent cases (labeled 0, 1, and 2) with a high viral load of Parvovirus B19. The dashed line indicates the 166bp peak that is observed for chromosomally derived cell-free DNA fragments.

Bimodal size distributions were observed for the other two betaherpesviruses, CMV (HHV5) and HHV7. The first peak of the distribution is observed at ~60bp, similar to mitochondrial cfDNA fragments. The second less pronounced peak is around 150bp, slightly shorter than chromosomal cfDNA, possibly indicating that both nucleosome-bound and unbound viral cfDNA are observed in blood plasma. For Bocaparvoviruses and Parvovirus B19, a similar bimodality can be observed [Figure 1]. Three samples with a (suspected) acute infection had an extraordinarily high viral load of Parvovirus B19, with between 3000 and 6000 viral reads per sample. By inspecting the fragment size distributions of these individual samples, we found that they varied between samples and that both short and long cfDNA fragments were present within all samples [Figure 3].

Finally, the size distribution of viral fragments from circular viruses [torque-tenovirus (TTV), human papillomavirus (HPV), hepatitis B virus (HBV), and Polyomaviruses] was significantly different from those of linear viruses (Adenovirus, AAV, HSV, VZV, CMV, HHV7, B19V, EBV) (Kolmogorov Smirnoff test, *P *= 1.68e-198). The median fragment size of circular viruses was 133bp, while the median fragment size of linear viruses was only 87bp. For TTV and Polyomavirus, fragment sizes are widely distributed, with slightly more fragments around the size of a single nucleosome. For HPV and HBV, we observe a random distribution of fragment sizes without any apparent increase in nucleosome-sized fragments, similar to alphaherpesviruses, adenoviruses, and mitochondrial cfDNA, but consisting of longer fragments.

## DISCUSSION

Fragmentation patterns of cell-free plasma DNA are essential biomarkers in the rapidly increasing field of liquid biopsies. Therefore, we studied the fragmentation patterns of viral plasma DNA, obtained from 83.552 mostly healthy pregnant women, and demonstrated specific variations between different viruses and presumably during specific infections.

In general, we observe different types of size distributions that can be related to different forms of viral infections compared with other cfDNA types. Size distribution of viral cfDNA fragments in the size range of 130bp to 170bp is comparable to cfDNA from chromosomal cfDNA where the size distribution is known to depend on nucleosome binding. As a consequence, this size distribution of viral cfDNA might be the result of nucleosomes that bind the viral DNA, presumably as part of the host innate immune response to inhibit viral gene expression, or because the viral DNA was integrated into the genomic DNA, as is the case for HHV6. In analogy with cfDNA fragmentation of nucleosome-free mitochondrial cfDNA, shorter viral cfDNA fragments are expected to stem from nucleosome-free viral DNA. Possible sources could be degraded virions or replicating DNA from active infections.

The bimodal distributions observed for a select subset of viruses might indicate that cell tropism, model of latency, and/or the immune response to these viruses are differently regulated. We hypothesize that, as different viruses target different cell-types and cell-types differentially contribute to the composition of cfDNA in plasma (a property used in tumor liquid biopsies to determine the cell-type of origin^[6]^), this may influence the number of the observed nucleosome bound viral fragments based on the viral species. For example, CMV, EBV, HHV6, HHV7, and Parvovirus B19 predominantly infect (and achieve latency) hematopoietic progenitor cells and leukocytes, which are all essential contributors to cfDNA in blood plasma. This contrasts to HPV, HBV, Adeno, VZV, and HSV, which mainly target epithelial, endothelial, and nerve cells, cell types that contribute less to the pool of circulating cfDNA in blood plasma, which might explain the presence of a nucleosome-bound peak in the first group, and its absence in the latter group of viruses.

Interestingly, in three cases with a high viral load of parvovirus B19 DNA, suggestive of an active or more recent infection, we observe that within these samples, both short (< 100bp) and long (nucleosome-sized) fragments co-occur and that the frequency with which they occur is highly variable between samples. We suspect that this could be representative of the number of circulating virions (short fragments) *vs.* the number of infected cells (nucleosome-sized fragments), which in turn may represent the differential timing of sampling within these active infections.

The size distribution of viral fragments originating from circular viruses was significantly different from that of linear viruses. The median fragment size from circular viruses was larger than that of linear viruses, possibly reflecting the protection of circular DNA from exonuclease activity^[1]^.

The observed variation in viral fragment sizes has consequences for the design of accurate quantitative PCR-based assays^[25]^. As many fragments are very small, primer-pairs used for PCR should not be widely spaced to prevent missing these fragments.

In this work, we found that on a per-sample basis, very little viral fragments were sequenced. Possibly this is due to the double-stranded library preparation protocol was used, as previous work has shown that single-stranded library preparation can significantly increase the number of sequenced viral fragments in plasma DNA. Future work should address whether our findings can be replicated with a single-stranded DNA library preparation protocol, as this could significantly improve the potential of viral plasma DNA sequencing on a per-sample basis.

In summary, we have shown that sequenced viral cfDNA fragments that were extracted from a large pool of NIPT sequencing data exhibit virus-specific size distributions, which are non-random and mostly distinct from typical chromosomal cfDNA. The mechanisms that give rise to these patterns are largely unknown, but our data link it to viral tropism, chromatinization, and the topology of the viral DNA. Further research is needed to evaluate how these characteristics of circulating viral cfDNA can be used in a clinical setting. Preliminary studies have been started in our center.

## DECLARATIONS

### Copyright

© The Author(s) 2021.
